# Purification and Preliminary Characterization of Tetraheme Cytochrome c_3_ and Adenylylsulfate Reductase from the Peptidolytic Sulfate-Reducing Bacterium *Desulfovibrio aminophilus* DSM 12254

**DOI:** 10.1155/BCA.2005.81

**Published:** 2005

**Authors:** Alejandro López-Cortés, Sergey Bursakov, Angelo Figueiredo, Anders E. Thapper, Smilja Todorovic, José J. G. Moura, Bernard Ollivier, Isabel Moura, Guy Fauque

**Affiliations:** 1 Centro de Investigaciones Biológicas del Noroeste (CIBNOR). Mar Bermejo 195 Playa Palo Santa Rita Baja California Sur La Paz 23090 Mexico; 2 REQUIMTE/CQFB Departamento de Quimica Faculdade de Ciências e Tecnologia da Universidade Nova de Lisboa Monte de Caparica 2829-516 Portugal; 3 Laboratoire de Microbiologie IRD UR 101, IFR-BAIM ESIL Universités de Provence et de la Méditerranée Campus de Luminy, Case 925 Marseille Cedex 09 13288 France

## Abstract

Two proteins were purified and preliminarily characterized from the soluble extract of cells (310 g, wet weight) of the aminolytic and peptidolytic sulfate-reducing bacterium *Desulfovibrio (D.) aminophilus* DSM 12254. The iron-sulfur flavoenzyme adenylylsulfate (adenosine 5'-phosphosulfate, APS) reductase, a key enzyme in the microbial dissimilatory sulfate reduction, has been purified in three chromatographic steps (DEAE-Biogel A, Source 15, and Superdex 200 columns). It contains two different subunits with molecular masses of 75 and 18 kDa. The fraction after the last purification step had a purity index (A_278nm_ / A_388nm_) of 5.34, which was used for further EPR spectroscopic studies. The *D. aminophilus* APS reductase is very similar to the homologous enzymes isolated from *D. gigas* and *D. desulfuricans* ATCC 27774. A tetraheme cytochrome c_3_ (His-heme iron-His) has been purified in three chromatographic steps (DEAE- Biogel A, Source 15, and Biogel-HTP columns) and preliminarily characterized. It has a purity index ([A_553nm_ - A_570nm_]_red_ / A_280nm_) of 2.9 and a molecular mass of around 15 kDa, and its spectroscopic characterization (NMR and EPR) has been carried out. This hemoprotein presents similarities with the tetraheme cytochrome c_3_ from *Desulfomicrobium (Des.) norvegicum* (NMR spectra, and N-terminal amino acid sequence).


### Purification and Preliminary Characterization of Tetraheme Cytochrome $c_3$ and Adenylylsulfate Reductase from the Peptidolytic Sulfate-Reducing Bacterium *Desulfovibrio aminophilus* DSM 12254

Alejandro López-Cortés[1] *, Sergey Bursakov[2], Angelo Figueiredo[2], Anders E.Thapper[2], Smilja Todorovic[2], José J.G. Moura[2], Bernard Ollivier[3], Isabel Moura[2] and Guy Fauque[3]

[1]*Centro de Investigaciones Biológicas del Noroeste, (CIBNOR). Mar Bermejo 195, Playa Palo Santa Rita, La Paz, Baja California Sur 23090, México.*
[2]*REQUIMTE/CQFB, Departamento de Quimica, Faculdade de Ciências e Tecnologia da Universidade Nova de Lisboa, 2829-516 Monte de Caparica, Portugal.*
[3]*Laboratoire de Microbiologie IRD, UR 101, IFR-BAIM, ESIL Universités de Provence et de la Méditerranée, Campus de Luminy, Case 925, 13288 Marseille Cedex 09, France.*

**ABSTRACT**

Two proteins were purified and preliminarily characterized from the soluble extract of cells (310 g, wet weight) of the aminolytic and peptidolytic sulfate-reducing bacterium *Desulfovibrio (D.) aminophilus* DSM 12254. The iron-sulfur flavoenzyme adenylylsulfate (adenosine 5′-phosphosulfate, APS) reductase, a key enzyme in the microbial dissimilatory sulfate reduction, has been purified in three chromatographic steps (DEAE-Biogel A, Source 15, and Superdex 200 columns). It contains two different subunits with molecular masses of 75 and 18 kDa. The fraction after the last purification step had a purity index ($A_{278\ nm}/A_{388\ nm}$) of 5.34, which was used for further EPR spectroscopic studies. The *D. aminophilus* APS reductase is very similar to the homologous enzymes isolated from *D. gigas* and *D. desulfuricans* ATCC 27774. A tetraheme cytochrome $c_3$ (His-heme iron-His) has been purified in three chromatographic steps (DEAE- Biogel A, Source 15, and Biogel-HTP columns) and preliminarily characterized. It has a purity index ($[A_{553\ nm}-A_{570\ nm}]_{red}/A_{280nm\ ox}$) of 2.9 and a molecular mass of around 15 kDa, and its spectroscopic characterization (NMR and EPR) has been carried out. This hemoprotein presents similarities with the tetraheme cytochrome $c_3$ from *Desulfomicrobium* (*Des.*) *norvegicum* (NMR spectra, and N-terminal amino acid sequence).

*Corresponding author: Alejandro López-Cortés. Phone: (612) 123-84-25 Fax: (612) 125-36-25

e-mail: alopczOA@cibnor.mx

**Abbreviations:** adenosine 5′-phosphosulfate or adenylylsulfate (APS), flavin adenine dinucleotide (FAD), adenosine monophosphate (AMP), diethylaminoethyl (DEAE), hydroxyapatite (HTP), electron paramagnetic resonance (EPR), nuclear magnetic resonance (NMR), polyacrylamide gel electrophoresis (PAGE), sodium dodecyl sulfate (SDS), 2,2-dimethyl-2-silapentane-5-sulfonate (DSS), ultraviolet (UV), *Desulfovibrio* (*D.*), *Desulfomicrobium* (*Des.*), Deutsche Sammlung von Mikroorganismen (DSM), Laboratoire de Chimie Bacterienne, (LCB), Centre National de la Recherche Scientifique (CNRS), basic local alignment search tool (BLAST), Dèpartement Soutien et Formation des Communautés Scientifiques du Sud (DSF), Institut de Recherche pour le Developpement, (IRD).

### INTRODUCTION

Sulfate-reducing bacteria constitute a group of anaerobic prokaryotes sharing the capacity to carry out dissimilatory sulfate reduction to sulfide as a major component of their bioenergetics processes [1-3], and contain a complex and diversified electron carrier system [4, 5]. *D. aminophilus* DSM 12254 is a mesophilic strain of sulfate-reducing bacterium isolated from an anaerobic sludge of a dairy wastewater treatment plant in Santa Fe de Bogota, Colombia [6]. This strain uses a wider range of energy substrates than reported for most *Desulfovibrio* species [1]. It presents, in particular, an important property of fermenting or oxidizing proteinaceous compounds, such as amino acids and peptides. This sulfate-reducing strain is also able to disproportionate sulfite and thiosulfate, suggesting that it plays a major role in regulating electron flow in the dissimilatory sulfur cycle [6]. We report here purification and preliminary characterization of two proteins involved in the respiratory system of *D. aminophilus*: one electron carrier, the tetraheme cytochrome $c_3$, and one enzyme, the adenylylsulfate reductase.

### EXPERIMENTAL

### Bacterial strain and growth conditions

*D. aminophilus* DSM 12254 was grown at 37°C in a lactate/sulfate medium under anaerobic conditions in the Unité de Fermentation, Laboratoire de Chimie Bacterienne (LCB), Centre National de la Recherche Scientifique (CNRS), in Marseille, France, and cells were harvested as previously described [7].

### Preparation of the soluble fraction

The cells (310 g, wet weight) were suspended in 10 mM Tris-HCl buffer, pH 7.6, then ruptured by passing twice through a French press. The extract was centrifuged for 1 h at 15,000 g and the supernatant (crude cell extract.) was centrifuged for 40 min at 26,000 g to separate the membrane (pellet) from the soluble extract.

### Proteins purification

The soluble fraction was then loaded onto a DEAE Bio-Gel A column (Bio-Rad, 44 x 4.5 cm) equilibrated with 10 mM Tris-HCl buffer, pH 7.6. A gradient of 1l 10mM Tris-HCl pH 7.6 and 1l 500 mM Tris-HCl pH 7.6 was set up. Five major proteins were eluted from the column: tetraheme cytochrome $c_3$, two molybdenum iron-sulfur-containing proteins (one with aldehyde oxidoreductase activity), bisulfite reductase of the desulfoviridin-type, and adenylylsulfate reductase. Two of these proteins (tetraheme cytochrome $c_3$ and APS reductase) were completely purified in two supplementary purification steps. The tetrahemic cytochrome $c_3$ fraction, after concentration on a Diaflo apparatus using a YM-10 membrane, was then applied to a Pharmacia Biotech ion exchange column Source 15 (32 x 2.6 cm) equilibrated with 10 mM Tris-HCl pH 7.6 and eluted with 10 mM Tris-HCl pH 7.6 to 10 mM Tris-HCl pH 7.6 and 500 mM NaCl. After concentration (Diaflo YM-10 membrane), the tetraheme cytochrome $c_3$ was finally passed over a Biogel-HTP column (45 x 1.6 cm) equilibrated with 10 mM Tris-HCl pH 7.6 and eluted with a continuous gradient of sodium phosphate buffer pH 7.6 (250 ml 10 mM/250 ml 250 mM). After concentration (Diaflo YM-30 membrane), the APS reductase fraction eluted from the DEAE-Biogel A column was applied to a Source 15 column (Pharmacia Biotech, 32 x 2.6 cm) equilibrated with 10 mM Tris-HCl buffer pH 7.6 and eluted with 10 mM Tris HCl pH 7.6 to 10 mM Tris-HCl + 500 mM NaCl. APS reductase after concentration (Diaflo YM-30 membrane) was finally passed over a Superdex 200 column (Amersham Biosciences, 67 x 2.6 cm) equilibrated with 50 mM Tris-HCl pH 7.6 and 300 mM NaCl.

### Molecular mass and purity determination

Subunit composition, molecular mass, and purity of proteins were determined by denaturing PAGE, using as running buffer Tris (0.025M)-glycine (0.192M), SDS (0.1%) pH 8.3. Low molecular weight kit markers for SDS electrophoresis (Pharmacia Biotech 17-0446-01) were used for the calibration of APS reductase and tetraheme cytochrome $c_3$. The protein standards with approximate molecular weights were: phosphorylase b, 94 kDa; albumin, 67 kDa; ovoalbumin 43 kDa; carbonic anhydrase 30 kDa; trypsin inhibitor 20.1 kDa and alfa lactalbumin 14.4 kDa. The gels were stained for protein by coomassie blue 0.5%.

### Ultraviolet (UV)-visible spectroscopy

UV-visible absorption spectra were recorded on a Shimadzu UV-2101 PC split beam spectrophotometer using 1-cm quartz cells.

### Electron paramagnetic resonance (EPR) spectroscopy

EPR spectra were recorded on a X-band Bruker EMX spectrometer equipped with a dual-mode cavity (Model ER 4116DM). Samples were cooled with helium gas using a continuous-flow cryostat (Oxford Instruments, UK).

### Nuclear magnetic resonance (NMR) spectroscopy

The NMR spectra were taken for a 0.8-1.0 mM tetraheme cytochrome $c_3$ sample in $D_2O$. The NMR spectra of *D. aminophilus* tetraheme cytochrome $c_3$ in the oxidized state were recorded on a 400 MHz Bruker ARX-400 spectrometer equipped with an inverse detection 5-mm probe and a variable temperature unit Bruker B-VT 2000. The 1D-NMR spectra were measured in oxidized state at 317°K, 313°K, 308°K, 303°K, and 283.2°K (pH 7.6) with a spectral width of 40.3kHz and a transmitter power level of 1.0 dB. All experiments were obtained with water pre-saturation and chemical shift and presented in ppm relative to the internal standard 2,2-dimethyl-2-silapentane-5-sulfonate (DSS).

### N-terminal amino acid sequencing of tetraheme cytochrome $c_3$

N-terminal amino acid sequence of *D. aminophilus* tetraheme cytochrome $c_3$ was determined by automated Edman degradation in a protein sequencer (Applied Biosystem model 477) coupled to an analyzer (Applied Biosystem model 120) following the manufacturer's instructions, using 100 pmol of tetraheme cytochrome $c_3$.

### RESULTS AND DISCUSSION

### Adenylylsulfate (APS) reductase

Although the first reports on APS reductase were published in the sixties, its three-dimensional structure was published only recently [8]. Comparison of physicochemical and spectroscopical properties of APS reductases isolated from several *Desulfovibrio* species show great similarity and high degree of homology [9]. Only recently has APS reductase been isolated from *Archaeoglobus fulgidus* and found to be a heterodimer with one subunit (75 kDa, 1FAD) and one subunit (18 kDa, 2 [4Fe-4S]) [10]. APS reductase has been purified in three chromatographic steps from the soluble fraction of *D. aminophilus* and preliminarily characterized. The UV-visible spectrum of APS reductase in the native form shows a broad maximum around 392 nm with shoulders at 445 and 475 nm and a protein absorption peak at 278 nm (unpublished results). The overall visible spectrum indicates the presence of a flavin group and iron sulfur centers. APS reductase is a heterodimer with one subunit (75 kDa, containing FAD) and one subunit (18 kDa, containing 2[4Fe-4S] centers) (Fig. 1).

EPR spectroscopic studies were carried out at different temperatures with the *D. aminophilus* APS reductase in the native state (Fig. 2-A) in the presence of natural substrates (AMP and sulfite) (Fig. 2B) and in the reduced form (Fig. 2C and 2D). Temperature dependence studies helped separate resonances originating in different species. In the native state (Fig. 2A), two clusters of APS reductase are in the $[4Fe\text{-}4S]^{2+}$ oxidized state, with four iron atoms as $Fe^{2.5+}$, giving total spin of S = 0. Nevertheless, the EPR spectrum shows a signal spread around g = 2.00. The spectral shape and g value of the signal indicate that the broad resonance accounts for only 0.1-0.25 spins/mol, which can be attributed to the residual [3Fe-4S] cluster [9]. This signal is in *D. aminophilus*, superimposed with the FAD radical (g = 2.0048). As seen in Fig. 2, the

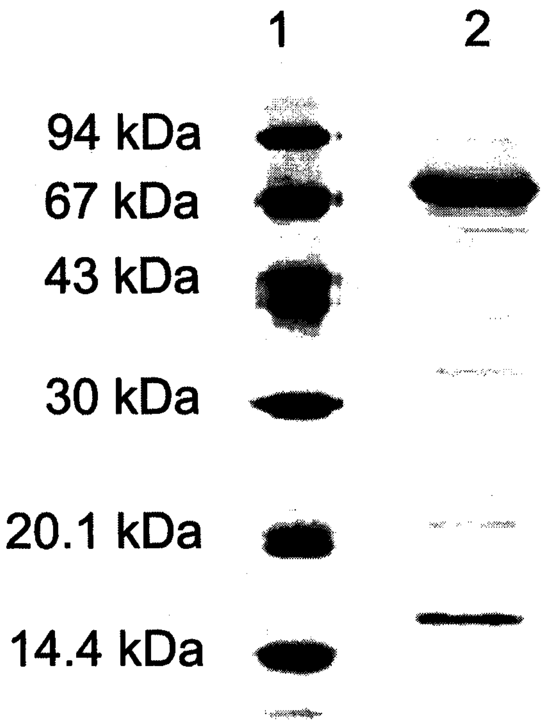

**Fig. 1:**`Denaturing SDS-PAGE 12.5%. Lane 1: profile of low molecular-weight markers. Lane 2: a heterodimer APS from *D. aminophilus* reductase with one subunit, around 75 kDa, containing FAD, and another subunit visible, around 18 kDa, from *D. aminophilus.*

former resonance can be detected up to ~30°K and the latter up to 45°K. The short reduction of the protein with $Na_2S_2O_4$ (at 15 sec, pH 9.5) (Fig. 2C), as well as the addition of the substrates AMP and $Na_2SO_3$ (Fig. 2-B), gives rise to a rhombic signal (g1 = 2.084, g2 = 1.94 and g3 = 1.90) that was attributed to the reduced $[4Fe\text{-}4S]^+$ Center I, S = 1/2. In addition to the iron-sulfur cluster resonances, there was a g = 2.0048 signal originating from the FAD radical and the residual resonance of the native APS reductase. The studies of temperature and power dependence of the spectra indicate that the FAD radical is present up to 100°K (data not shown), while the Center I cluster can be seen up to 45°K. Apparently, the long reduction with $Na_2S_2O_4$ (Fig. 2D) did not result in a fully reduced APS reductase after several attempts to fully reduce the sample. After more than 30 min of reduction with dithionite (pH 9.5), both [4Fe-4S] clusters should be reduced, each having total spin S = 1/2, but the EPR spectrum of APS reductase most likely originates from only one reduced [4Fe-4S]. It is not clear at this point whether this can be related to some specific characteristic of APS reductase in *D. aminophilus*.

APS reductase is a major cytoplasmic enzyme constituting 2 to 3% of soluble proteins in sulfate reducers of the genus *Desulfovibrio* [4, 5]. It is a nonheme iron flavoprotein which has also been found in several genera of sulfate-reducing bacteria: *Desulfobacter, Desulfotomaculum, Desulfosarcina, Desulfococcus, Desulfobulbus, Thermodesulfobacterium,* and *Archaeoglobus* [4, 9, 10, 11]. APS reductase from eight species and strains of *Desulfovibrio* and one from *Archaeoglobus* were purified and their biochemical and

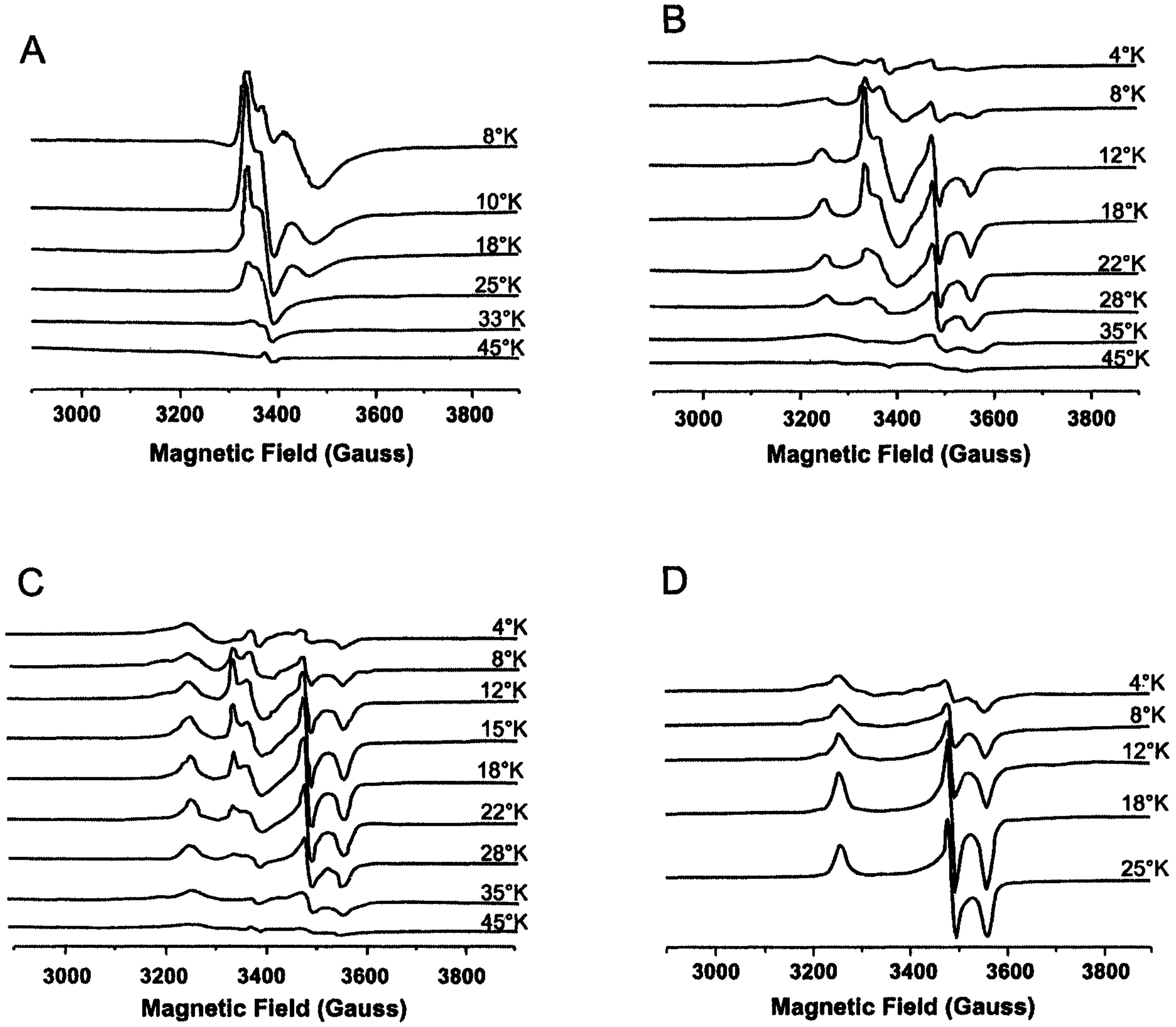

**Fig. 2:** Temperature dependece of EPR spectra of APS reductase from *D. aminophilus* with: (A) Native enzyme, signal from residual [3Fe-4S] cluster. (B) native APS reductase incubated with substrates AMP and $SO_3^{-2}$. (C) native APS reductase with $Na_2S_2O_4$ t ~15 sec (only Center I is reduced). (D) APS reductase with $Na_2S_2O_4$ t > 30 min (both [4Fe-4S] clusters are reduced). The spectrometer conditions were: microwave power: 2 mW; microwave frequency: 9.485 GHz; modulation amplitude: 8 G; modulation frequency: 100 kHz; field center: 3400 G; sweep width: 1000 G.

spectroscopic properties determined. They present a high degree of homology in their physicochemical characteristics and their visible and EPR spectra [9]. APS reductases isolated from *Desulfovibrio* species are proteins containing one FAD per molecule and eight iron atoms arranged in two [4Fe-4S] clusters (Center I and Center II). They have a monomeric molecular mass ranging between 150 and 180 kDa and possess two different subunits with molecular masses of around 20 and 70 kDa [9]. The reaction of sulfite with APS reductase results in the formation of a FAD-sulfite adduct causing the bleaching of the FAD and the appearance of a maximum at 320 nm, corresponding to the reaction of sulfite at the N-5 position of the isoalloxazine ring of FAD. The subsequent addition of AMP results in a decrease in absorbance at 320 nm, partial reduction of iron-sulfur centers, and the formation of APS. A common feature of all APS reductases from *Desulfovibrio* species is the perturbation of the EPR spectral features of Center I after its reaction with AMP and sulfite, as well as its high redox potential (0 to –50 mV) when compared with other [4Fe-4S] clusters. Center II is a [4Fe-4S] cluster with a redox potential lower than –400 mV [9]. APS reductase in sulfate-reducing bacteria is an enzyme highly conserved in terms of its composition at the active site as well as its physiological properties.

### Tetrahemic cytochrome $c_3$

A tetrahemic cytochrome $c_3$ (His-heme iron-His) has been purified in three chromatographic steps from the *D. aminophilus* soluble extract. It has a purity index ($[A_{553\ nm}-A_{570\ nm}]_{red}/A_{280\ nm\ ox}$) equal to 2.90. Denaturing SDS-PAGE corroborated the purity of this protein (Fig. 3). The UV-visible spectrum of the oxidized tetrahemic cytochrome $c_3$ exhibits a broad absorption band around 531 nm (beta band), a Soret peak (gamma band) with a maximum at 410 nm, another broad band at 350 nm (delta band), and a protein peak at 280 nm (Fig. 4). The tetraheme cytochrome $c_3$ is not reduced by sodium ascorbate, but is fully reduced by

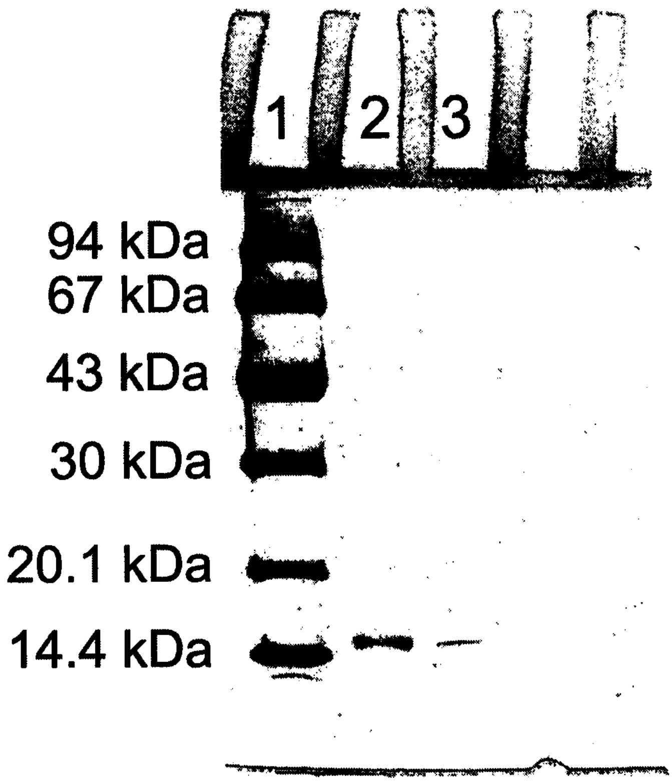

**Fig. 3:** Denaturing SDS-PAGE 15%. Lane 1: profile of low molecular-weight markers. Lane 2: tetrahemic cytochrome $c_3$ (5 μl). Lane 3: tetrahemic cytochrome $c_3$ (2 μl) from *D. aminophilus*; single band around 15 kDa/subunit interpreted as a pure protein.

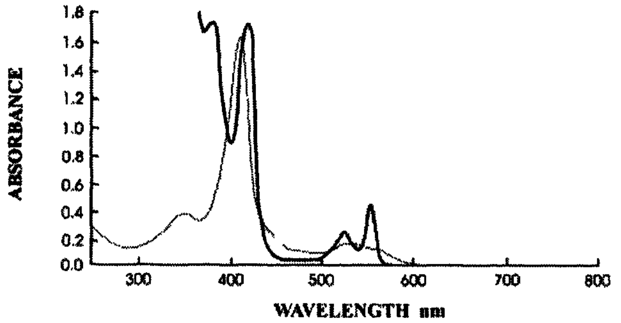

**Fig. 4:** UV-visible absorption spectra of *D. aminophilus* tetraheme cytochrome $c_3$ in the oxidized (thin line) and reduced (thick line) forms.

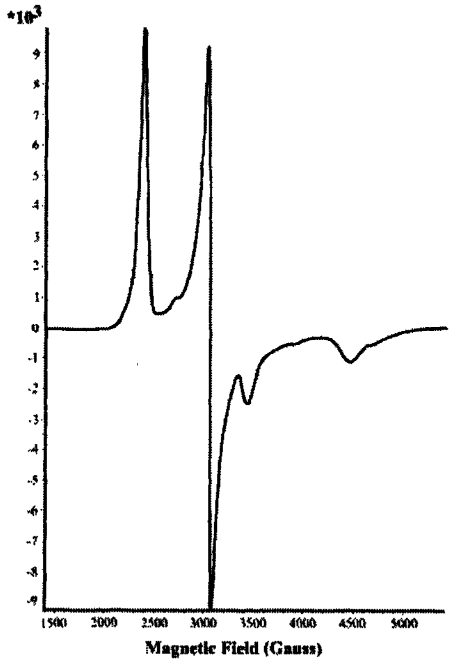

**Fig. 5:** EPR spectrum of *D. aminophilus* ferritetraheme cytochrome $c_3$. Conditions of measurement were: temperature10°K, microwave power: 2 mW; microwave frequency: 9.652 GHz; modulation amplitude: 10 G; modulation frequency: 100 kHz; field center: 3400 G; sweep width 4000 G.

sodium dithionite, showing absorption maxima at 553 nm (alpha band), 523 nm (beta band), and a Soret peak at 418 nm (gamma band) (Fig. 4). Figure 5 shows the EPR spectrum of the *D. aminophilus* ferritetraheme cytochrome $c_3$ recorded at 10°K. The spectrum shows a prominent feature at g = 2.920 in the g max region and a derivative peak is observed at g = 2.276 (probably g med). Figure 6 shows the NMR spectrum of the *D. aminophilus* ferritetraheme cytochrome $c_3$ recorded at 313°K. This NMR spectrum is very close to the one reported for *Des. norvegicum* tetraheme cytochrome $c_3$ [12] with two methyl resonances around 25 ppm and a single proton resonance well resolved at low field. N-terminal amino acid sequence of the *D. aminophilus*

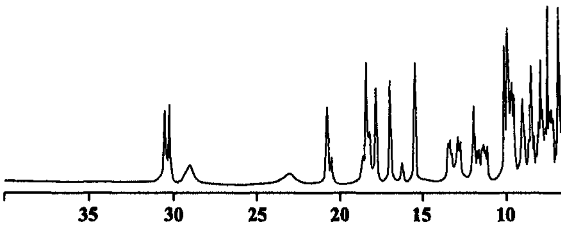

**Fig. 6:** NMR spectrum of *D. aminophilus* ferritetraheme cytochrome $c_3$ (concentration 0.8-1.0 mM, pH 7.6) recorded at 313°K. Conditions of measurement were: spectral width 40.3 kHz and a transmitter power level 1.0 dB.

*D. aminophilus* (1-32): 1 ADAPKQDIV-----MKS-----PGAXAQGTVTLSHAKH GGQQ 32

DSM12254 **ADAP D V MK+ PGA Q TV H KH**

*Des. norvegicum* (1-39): 1 ADAPGDDYVISAPEGMKAKPKGDKPGA-LQKTVPFPHTKH 39
P00136

**Fig. 7:** N-terminal amino acid sequencing of tetraheme cytochrome $c_3$ from *D. aminophilus* DSM 12254. This amino acid sequence was compared with other tetraheme cytochrome $c_3$ sequences using BLAST. Results showed a significant alignment with the primary structure of the homologous protein, tetraheme cytochrome $c_3$ of 13 kDa, GenBank accession number P00136 from *Des. norvegicum*. Identities = 17/40 (42%), Positives = 18/40 (44%), Gaps = 13/40 (32%).

tetraheme cytochrome $c_3$ was determined up to residue 32 (Fig. 7). This sequence was investigated with the protein Basic Local Alignment Search Tool software (BLAST), and showed a significant alignment with a tetraheme cytochrome $c_3$ of 13 kDa, with 118 residues (GenBank accession number P00136 from *Des. norvegicum* DSM 1741, formerly called *D. desulfuricans* strain Norway 4).

Tetraheme cytochrome $c_3$ is the only hemoprotein present in large amounts in all *Desulfovibrio* species so far isolated, and it is characteristic of this genus although it has also been found in two *Thermodesulfobacterium* species, *Des. norvegicum* and *Desulfobulbus elongatus* [4, 5]. Tetraheme cytochrome $c_3$ is a small (14 kDa) monomeric protein located in the periplasmic space that plays an important role in the metabolism of dissimilatory sulfate reduction [4, 5]. No unequivocal physiological function has been clearly established for tetraheme cytochrome $c_3$ even if it can act as a sulfur reductase in several strains of *Desulfovibrio* and *Desulfomicrobium* [13]. The four hemes of tetraheme cytochromes $c_3$ hemes have histidine-histidine ligation, and as shown by EPR and NMR spectroscopies, they are localized in non-equivalent protein environments, where each heme has a different redox potential value ranging from –50 to –400 mV [4, 5, 14]. EPR spectra of tetraheme cytochromes $c_3$ show features of four different low-spin Fe(III) hemes with bis-histidinyl co-ordination [15,16]. To date, the three-dimensional structures of seven tetraheme cytochrome $c_3$s have been determined by X-ray diffraction [16-19]. The most striking characteristics of the three-dimensional structures of the tetraheme cytochromes $c_3$ are the compact organization of the four hemes with a relatively high degree of solvent exposure. Despite the rather low homology among the amino-acid sequences of tetraheme cytochromes $c_3$ (lowest homology of 20%), no significant differences in the overall structure and spatial arrangement of the four hemes have been observed [16-19].

Here, we described the purification and preliminary characterization of two key proteins involved in the dissimilatory sulfate reduction pathway of *D. aminophilus*. We have shown that, according to the UV-visible and EPR spectra, *D. aminophilus* APS reductase is very close to the homologous enzymes isolated from *D. gigas* and *D. desulfuricans* ATCC 27774. *D. aminophilus* tetraheme cytochrome $c_3$ presents more homology with the homologous protein present in *Des. norvegicum* (N-terminal amino acid sequence and NMR spectra).

### ACKNOWLEDGEMENTS

We are indebted to R. Toci and M. Bàuzan for growing the bacteria used in this study. A. López-Cortés received financial support from the Département Soutien et Formation des Communautés Scientifiques du Sud (DSF)-Institut de Recherche pour le Développement (IRD), France, during his postdoctoral stay in Marseille, France. Editorial staff at CIBNOR improved the English text.

### REFERENCES

1. F. Widdel, In: A.J.B. Zehnder (Ed), *Biology of Anaerobic Microorganisms*, John Wiley & Sons, Inc., New York, 469 (1988)
2. G. D. Fauque, In: L.L. Barton (Ed.), *Biotechnology Handbooks*, Volume 8, *Sulfate-Reducing Bacteria*, Plenum Press, New York and London, 217 (1995)
3. G. Fauque and B. Ollivier, In: A. T. Bull (Ed), *Microbial Diversity and Bioprospecting*, ASM Press, Washington, D.C., 169 (2004)
4. J. LeGall and G. Fauque, In: A.J.B. Zehnder (Ed), *Biology of Anaerobic Microorganisms*, John Wiley & Sons, Inc., New York, 587 (1988)
5. G. Fauque, J. LeGall and L.L. Barton, In: J.M. Shively and L.L. Barton (Ed.), *Variations in Autotrophic Life*, Academic Press Limited, London, 271 (1991)
6. S. Baena, M.L. Fardeau, M. Labat, B. Ollivier, J.L. Garcia and B.K.C. Patel, *System. Appl. Microbiol*, **21**, 498 (1998)
7. J. LeGall, G. Mazza and N. Dragoni, *Biochim. Biophys. Acta*, **99**, 385 (1965)
8. G. Fritz, A. Roth, A. Schiffer, T. Buchert, G. Bourenkov, H.D. Bartunik, H. Huber, K.O Stetter, P.M.H. Kroneck, and U. Erlmer, *Proc. Nat. Acad. Sci*, **99**, 1836 (2002)
9. J. Lampreia, A.S. Pereira and J.J.G. Moura, *Methods in Enzymol*, **243**, 241 (1994)
10. G. Fritz, T. Buchert, H. Huber, K.O. Stetter and P.M.H. Kroneck, *FEBS Lett*, **473**, 63 (2000)

11. J. Lampreia, G. Fauque, N. Speich, C. Dahl, I. Moura, H.G. Truper and J.J.G. Moura, *Biochem. Biophys. Res. Commun.*, **181** , 342 (1991)
12. I. Moura, A.V. Xavier, J.J.G. Moura, G. Fauque, J. LeGall, G.R. Moore and B.H. Huynh, *Rev. Port. Quim.*, **27**, 212 (1985)
13. G.D. Fauque, *Methods in Enzymol.* **243**, 353 (1994)
14. I.B. Coutinho and A.V. Xavier, *Methods in Enzymol.* **243**, 119 (1994)
15. I. Moura, G. Fauque, J. LeGall, A.V. Xavier and J.J.G. Moura, *Eur. J. Biochem.*, **162,** 547 (1987)
16. O. Einsle, S Foerster, K. Mann, G. Fritz, A. Messerschmidt and P.M.H. Kroneck, *Eur. J. Biochem.*, **268,** 3028 (2001)
17. Y. Higuchi, H Akutsu and N Yasuoka, *Biochimie*, **76**, 537 (1994)
18. J. Morais, P.N. Palma, C. Frazao, J. Caldeira, J. LeGall, I. Moura, J.J.G. Moura and M.A. Carrondo, *Biochemistry*, **34**, 12830 (1995)
19. S. Norager, P. Legrand, L. Pieulle, C. Hatchikian and M. Roth, *J. Mol. Biol.*, **290**, 881 (1999)

